# Symbiont Community Composition in *Rimicaris kairei* Shrimps from Indian Ocean Vents with Notes on Mineralogy

**DOI:** 10.1128/aem.00185-22

**Published:** 2022-04-11

**Authors:** Pierre Methou, Masanari Hikosaka, Chong Chen, Hiromi K. Watanabe, Norio Miyamoto, Hiroko Makita, Yoshio Takahashi, Robert G. Jenkins

**Affiliations:** a X-STAR, Japan Agency for Marine-Earth Science and Technology (JAMSTEC), Yokosuka, Japan; b College of Science and Engineering, Kanazawa University, Kanazawa, Japan; c Department of Ocean Sciences, Tokyo University of Marine Science and Technology, Tokyo, Japan; d Department of Earth and Planetary Science, Graduate School of Sciences, The University of Tokyo, Tokyo, Japan; e Photon Factory, Institute of Materials Structure Science (IMSS), High Energy Accelerator Research Organization (KEK), Tsukuba, Ibaraki, Japan; University of Queensland

**Keywords:** Central Indian Ridge, chemosynthetic symbioses, decapod crustaceans, epibiosis, hydrothermal vent, microbialite

## Abstract

Hydrothermal vent ecosystems are home to a wide array of symbioses between animals and chemosynthetic microbes, among which shrimps in the genus Rimicaris is one of the most iconic. So far, studies of *Rimicaris* symbioses have been restricted to Atlantic species, including Rimicaris exoculata, which is totally reliant on the symbionts for nutrition, and the mixotrophic species Rimicaris chacei. Here, we expand this by investigating and characterizing the symbiosis of the Indian Ocean species Rimicaris kairei using specimens from two vent fields, Kairei and Edmond. We also aimed to evaluate the differences in mineralogy and microbial communities between two cephalothorax color morphs, black and brown, through a combination of 16S metabarcoding, scanning electron microscopy, fluorescent *in situ* hybridization, energy-dispersive X-ray spectroscopy, and synchrotron near-edge X-ray absorption structure analyses. Overall, our results highlight that R. kairei exhibits similar symbiont lineages to those of its Atlantic congeners, although with a few differences, such as the lack of Zetaproteobacteria. We found distinct mineralization processes behind the two color morphs that were linked to differences in the vent fluid composition, but the symbiotic community composition was surprisingly similar. In R. exoculata, such mineralogical differences have been shown to stem from disparity in the microbial communities, but our results indicate that in *R. kairei* this is instead due to the shift of dominant metabolisms by the same symbiotic partners. We suggest that a combination of local environmental factors and biogeographic barriers likely contribute to the differences between Atlantic and Indian Ocean *Rimicaris* symbioses.

**IMPORTANCE** Hydrothermal vent shrimps in the genus *Rimicaris* are among the most charismatic deep-sea animals of Atlantic and Indian Oceans, often occurring on towering black smokers in dense aggregates of thousands of individuals. Although this dominance is only possible because of symbiosis, no study on the symbiosis of Indian Ocean *Rimicaris* species has been conducted. Here, we characterize the *Rimicaris kairei* symbiosis by combining molecular, microscopic, and elemental analyses, making comparisons with those of the Atlantic species possible for the first time. Although most symbiotic partners remained consistent across the two oceans, some differences were recognized in symbiont lineages, as well as in the mechanisms behind the formation of two color morphs with distinct mineralogies. Our results shed new light on relationships among mineralogy, environmental factors, and microbial communities that are useful for understanding other deep-sea symbioses in the future.

## INTRODUCTION

Ecosystems on Earth were initially considered to be universally dependent on photosynthetic primary production, but a series of discoveries in the late 20th century, starting with deep-sea hydrothermal vents, unveiled a number of systems that rely on a different source of energy production—chemosynthesis (1). Hydrothermal vents are home to dense faunal communities sustained by chemosynthetic microbial activities that utilize the chemical energy from vent fluid emissions. The most successful animals inhabiting these ecosystems form intricate symbiotic relationships with chemosynthetic microorganisms, harboring various degrees of internalization that range from the simple settlement on specific body surfaces in ectosymbioses to the colonization of specialized internal organs in endosymbioses (2, 3). These symbionts are fueled by a wide range of energy sources, including a variety of sulfur compounds, methane, hydrogen, or even iron (4–7).

This diversity of metabolism mirrors a diversity of partners, mostly in Campylobacterota and Gammaproteobacteria (2), but more rarely also in Zetaproteobacteria or Desulfobacterota (2, 8). Except in some molluscs such as vesicomyid clams (9), the majority of these chemosymbioses are formed through horizontal transmission in which each generation of settling juveniles obtains its symbionts from the environment anew (10). Despite a certain level of partner fidelity between such hosts and their symbionts, symbioses established through horizontal transmission remain dynamic and flexible—the microbial community composition and function can shift according to various environmental factors, most importantly the composition of reduced compounds in the vent fluid (5, 11–13). These variations are all the more important in epibioses, where significant shifts in symbiotic community compositions according to variable fluid chemistries in different vent fields have been observed in several species (14, 15).

To fuel their symbiosis and meet the requirements of their chemosynthetic metabolisms, host animals at vents must also face the environmental stress of heavy metals found in high concentrations within these fluids. As a consequence, these organisms are often associated with abundant mineral deposits, on and/or within their body, and they are sometimes even involved in biomineralization processes (16–18). An emblematic example of such biomineralization is the scaly-foot snail (Chrysomallon squamiferum), the only known metazoan with a skeleton made of iron sulfide, which is formed by waste sulfur compounds produced by its endosymbionts reacting with iron ions from the vent fluid (17). Here, again, the nature of these biomineralizations differs according to vent fluid compositions, with scales ranging from black, brown, or white (in order of reducing iron content) in vent fields with contrasting iron concentrations (17, 19).

Among vent-endemic fauna, the alvinocaridid shrimp *Rimicaris exoculata* is probably one of the best-documented symbioses for chemosynthetic epibiosis (20). Within its enlarged branchiostegites, i.e., the inner parts of the carapace, and on its hypertrophied mouthparts, this shrimp hosts a diverse and dense symbiotic community of filamentous bacteria (14, 21–23). These symbionts provide most of the nutrition for the host (24–26) apparently through a direct transtegumental (i.e., across the cuticle) transfer of organic carbon (4). Chemosynthetic metabolisms found in these symbiotic communities are diverse, including oxidation and dismutation of sulfur compounds and the oxidation of hydrogen, methane, or iron (8, 22, 23, 27, 28). This metabolic flexibility of the bacterial partners certainly has an important role in the ecological success of R. exoculata within Atlantic hydrothermal ecosystems, enabling these animals to colonize a wide range of vent fields with contrasting chemical profiles, all along the Mid-Atlantic Ridge (MAR), where they live in dense swarms close to the vent fluid emissions (20, 29, 30).

Aside from *R. exoculata*, symbiosis in other closely related alvinocaridid shrimps have received little attention compared to that given to other animal groups with chemosymbioses (2, 31–34). At present, the only other alvinocaridid shrimp with characterized cephalothoracic bacterial communities is Rimicaris chacei (35), which cooccurs with *R. exoculata* in several vent fields on the northern MAR (29). This limits our capacity to compare the symbiotic adaptation of different lineages within the family and how the symbiont population may be linked to environmental factors in other vents around the world—even recently discovered and poorly known groups like Kiwa squat lobsters have symbioses characterized in more species across a wider geographic area (15, 36, 37). An obvious target for exploring alvinocaridid symbiosis outside the MAR is Rimicaris kairei, the species phylogenetically sister to *R. exoculata* but occurring in Indian Ocean vent fields. These two sister species share many similarities, including reproductive features, a much-enlarged cephalothorax, and occurrence in dense swarms (38). Although a symbiosis-based nutrition has been assumed for R. kairei based on stable isotope ratios (39), no information about its symbiotic communities have been published.

Another consequence of this symbiosis is the observable deposition of minerals inside the cephalothorax of these shrimp, resulting partially from abiotic processes but also from the activity of their iron-oxidizing symbionts (18, 40, 41). Several observations have shown that the color of these mineral deposits within the *R. exoculata* cephalothorax differs both between distinct vent fields but also within the same vent field (14, 20, 41, 42) and that the two different colors, i.e., black/gray versus red/brown, are associated with varied symbiont community compositions (14). Both these mineral deposits and the symbiotic communities are renewed approximately every 10 days and reacquired after each molt (42), making this symbiosis a highly dynamic and complex association. Like *Rimicaris exoculata*, *R. kairei* inhabits numerous vent fields with contrasting fluid chemistries, such as Kairei, which is characterized by high hydrogen and hydrogen sulfide concentrations, and Edmond, also with a high concentration of hydrogen sulfide but is lower in hydrogen concentration and much richer in iron (43, 44). Hence, color and mineralization within the *R. kairei* cephalothoracic cavity may reflect the differences in the precipitation of reduced compounds according to differences in vent fluid compositions and/or their transformation by symbiotic partners with different metabolic pathways.

**FIG 1:**
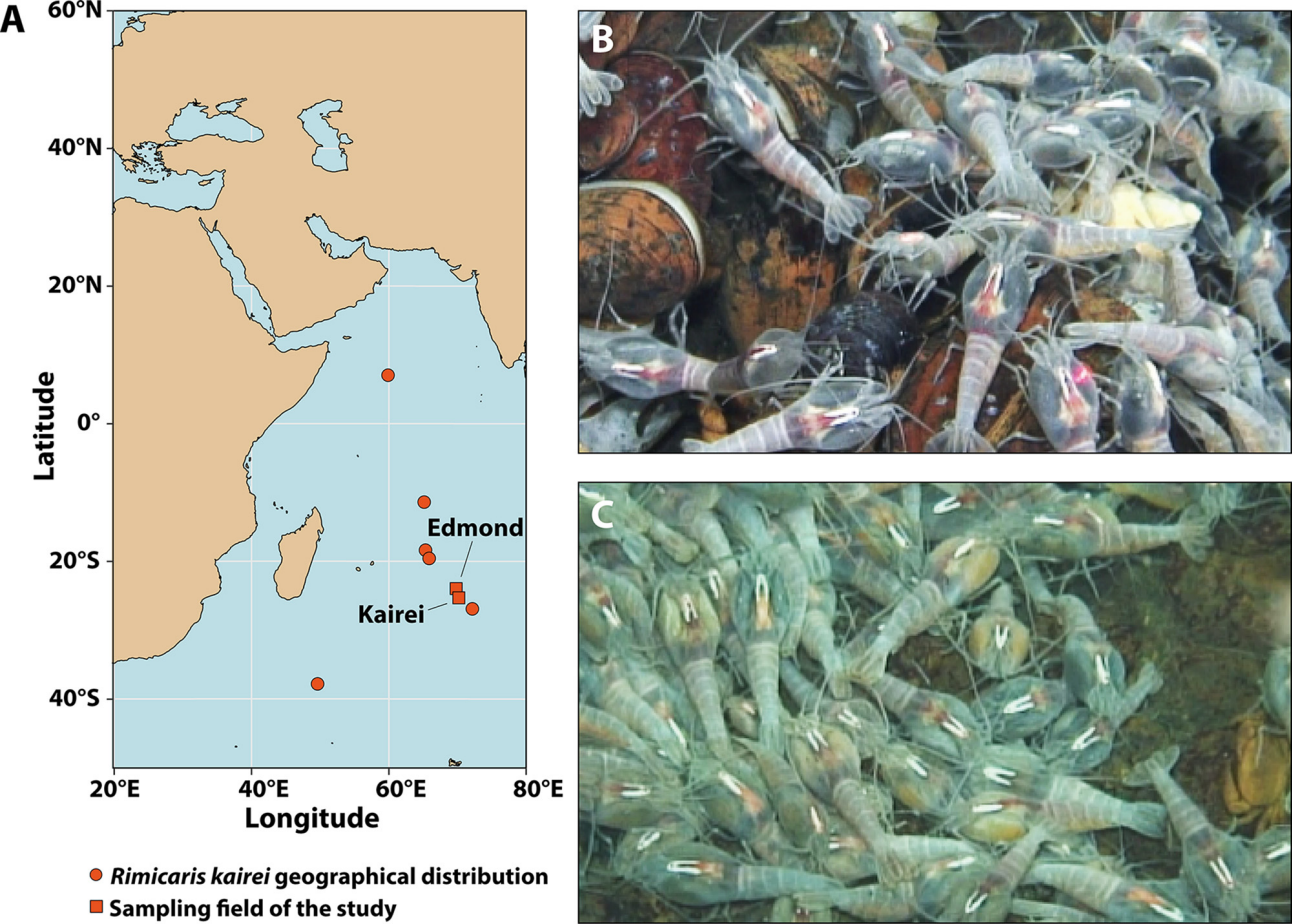
Overview of the study sites and *Rimicaris kairei* populations on the Central Indian Ridge. (A) Geographic location of the studied hydrothermal vent fields. (B) *R. kairei* dense swarms at Kairei, largely dominated by shrimp with a black cephalothorax. (C) *R. kairei* dense swarms at Edmond, dominated by shrimp with a brown cephalothorax.

Here, we provide the first investigation of the *R. kairei* symbiotic communities through a combination of 16S gene metabarcoding, scanning electron microscopy (SEM), and fluorescent *in situ* hybridization (FISH) observations. Furthermore, we were also able to collect *R. kairei* individuals in both black and brown color morphs from two vent fields (Fig. 1). We analyzed their mineralogical content in parallel using energy-dispersive X-ray spectroscopy (EDX) and X-ray absorption near edge structure (XANES) analyses to explore whether differences in cephalothorax color morphs and mineral contents reflect differences in the symbiotic communities of *R. kairei* and how they are impacted by fluid chemistry in the two vent fields.

## RESULTS

### Electron microscopy.

**FIG 2:**
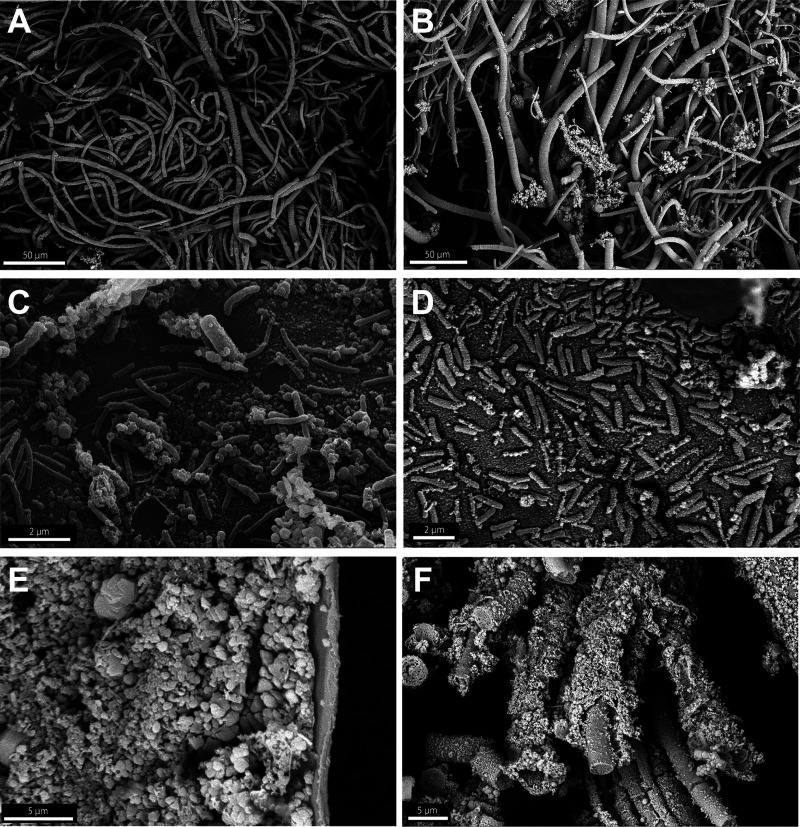
Scanning electron microscopy (SEM) observations showing epibiont microbe and mineral particles on the internal surface of branchiostegites from brown (A, C, E) and black (B, D, F) morphotypes of *Rimicaris kairei* from the Edmond and Kairei fields, respectively. (A, B) Aggregations of filamentous bacteria. (C, D) Rod-shaped bacteria on the surface of branchiostegites. (C) Some clusters of mineral particles were visible in brown morph shrimps (arrows). (E) Aggregations of nano- to 1-μm-sized mineral particles formed crusts on the internal surface of branchiostegites. (F) Sheath on filamentous bacteria in a black morph shrimp.

Observations of *Rimicaris kairei* branchiostegites under SEM showed a dense microbial colonization of filamentous bacteria comprising thick and thin morphotypes, mostly on anterior parts, in both brown (Fig. 2A) and black morphotypes (Fig. 2B). More posteriorly at the edge of this microbial colonization, we also observed a monolayer of coccoid and rod-shaped bacteria attached to the surface of the branchiostegites (Fig. 2C and D). These microbial communities were found to be associated with mineral deposits, which formed as a crust on the basal part of the branchiostegites in the brown morphotype (Fig. 2E) and occurred as a sheath of mineral particles embedding the filamentous bacteria in the black morphotype (Fig. 2F). In contrast, the posterior part of the branchiostegites, corresponding to the part facing the gills, was deprived of bacterial colonization and mineral deposits in both morphotypes (see Fig. S1 in the supplemental material).

### Elemental and mineralogical analyses.

Elemental analysis of the particle deposits on *R. kairei* branchiostegites using field emission SEM (FE-SEM) revealed the presence of iron, oxygen, silica, and calcium as major peaks in brown particles in the brown morphotype, and carbon, magnesium, phosphorus, and calcium as major peaks and sulfur and iron as minor peaks in black particles on/in a sheath of filamentous bacteria in the black morphotype (Fig. S2). A much stronger S/Fe signal in the black morphotype than that in the brown morph indicates relatively large amount of sulfide in the black morph. More detailed elemental distribution maps obtained by synchrotron X-ray absorption fine structure (XAFS) spectroscopy show that many other metal elements are included in those particles (Fig. S3).

**FIG 3:**
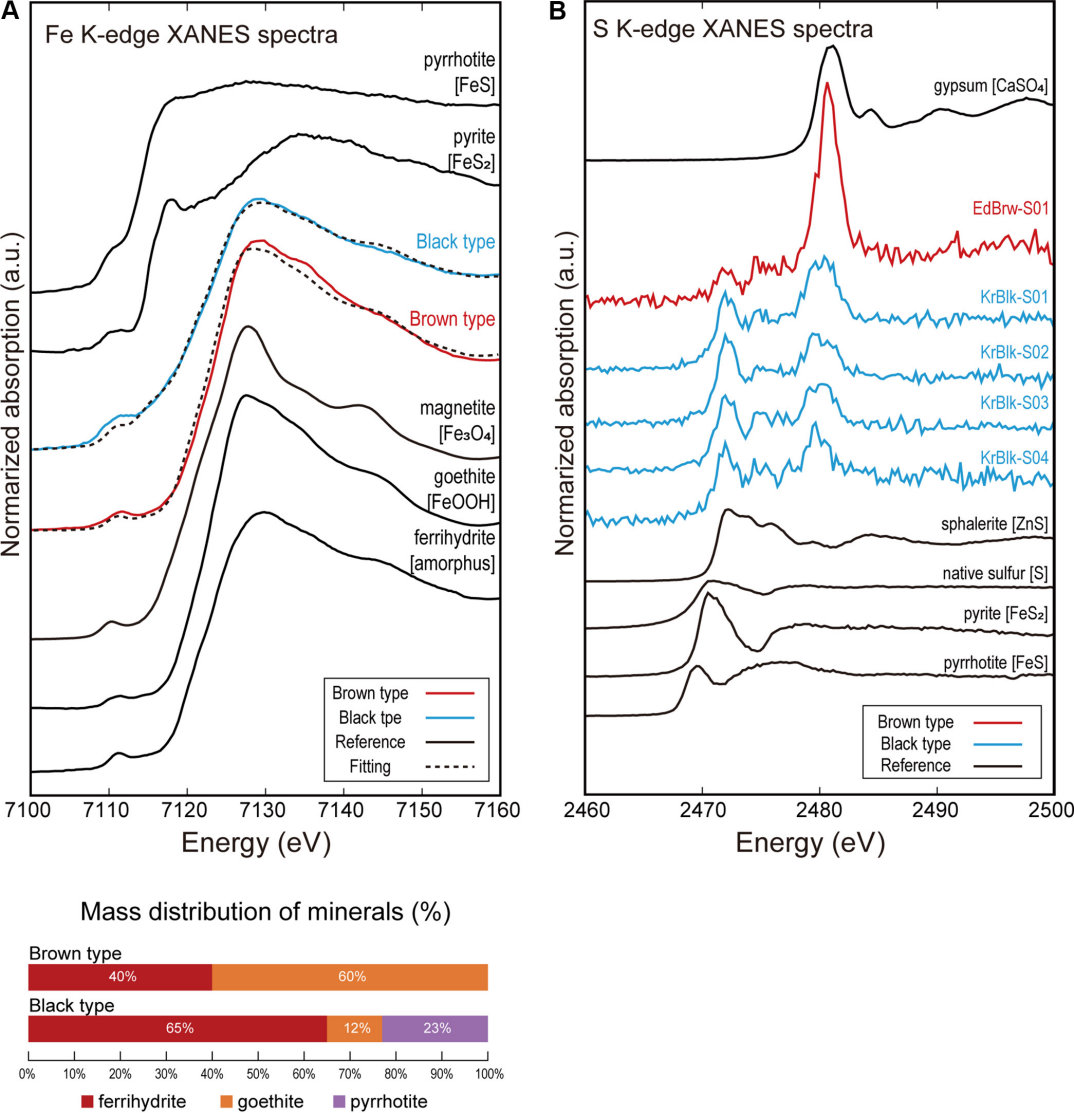
X-ray absorption near edge structure (XANES) spectra of brown and black mineral deposits on *Rimicaris kairei*. (A) Normalized Fe K-edge μ-XANES spectra of the brown and black shrimp (red and blue solid lines, respectively) and reference material (pyrrhotite, pyrite, magnetite, goethite, and ferrihydrite). The spectra were combined from several analytical points for both brown and black morphs. Results of fitting analysis are shown at the bottom. (B) Normalized S K-edge μ-XANES spectra of the brown and black morphs (red and blue solid lines, respectively) and reference materials (gypsum, sphalerite, native sulfur, pyrite, and pyrrhotite). Analytical points for the XANES spectra are shown in Fig. S4 in the supplemental material.

Fe K-edge μ-XANES spectra were used to determine the chemical species of Fe and showed an absorption peak around 7.128 to 7.130 keV and a pre-edge peak around 7.111 to 7.112 keV for the brown particles (Fig. 3A). These brown particles contained Fe(III) oxyhydroxides such as goethite and ferrihydrite. For the black particles, the absorption peak of the μ-XANES spectrum was around 7.128 to 7.130 keV, while the pre-edge peak was around 7.109 to 7.112 keV with a weak shoulder around 7.116 to 7.122 keV compared with the brown sample. The pre-edge peak was shifted to lower energy by 1 to 2 eV (Fig. 3). In addition, the shoulder and shape of the pre-edge peak suggest that pyrite (FeS_2_) and/or pyrrhotite (FeS) were included in the black particles. Fitting of the spectrum by linear combination of reference materials showed that ca. 23% of the Fe chemical species in the black particles were pyrrhotite (a type of Fe sulfide).

S K-edge μ-XANES spectra were used to determine the chemical species of S and showed peaks at 2.480 keV for brown particles and at 2.472, 2.474, and 2.480 keV for black particles (Fig. 3B). The peak of the brown particles points to oxidized sulfur species such as sulfate as a main component of the brown morph. On the other hand, the black particles mainly comprised reduced sulfur species; however, the spectra did not fit perfectly with the reference material analyzed. The spectra of the black particles all exhibited a peak at 2,472.0 eV, 8.5 to 9 eV lower than the sulfate peak of organosulfur species such as thiol (R-SH) and sulfide (R-S-R′) groups (45, 46), although we could not compare directly to the sulfur-containing organic molecules because we did not analyze reference material of those organic molecules.

Overall, the elemental and mineralogical analyses indicated that the brown particles in *R. kairei* branchiostegites were composed of Fe oxides, while the black particles contained moderate amounts of a mixture of iron sulfide (or other reduced sulfur minerals) and thiol or sulfide (R-S-R′) with a large amount of Fe oxides.

### Microbial diversity analyses.

**FIG 4:**
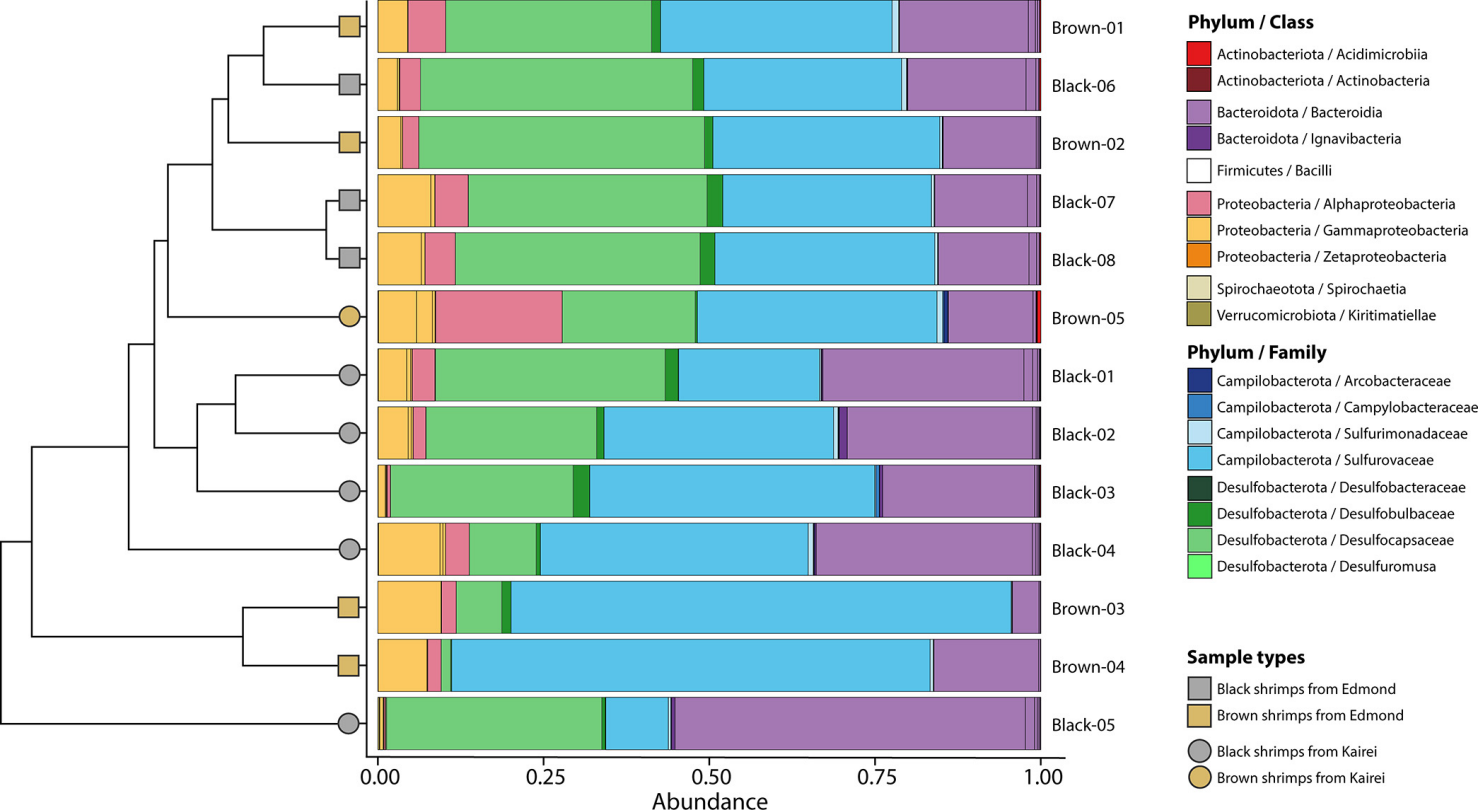
Relative abundances of 16S rRNA gene sequence reads from *Rimicaris kairei* branchiostegites according to their classification (Silva 138 database). Groups are at the family level for the *Campylobacterota* and *Desulfobacterota* phyla and at the class level for other phyla. The cluster dendrogram depicts the average linkage hierarchical clustering based on a Bray-Curtis dissimilarity matrix of community compositions resolved down to amplicon sequence variant (ASV) level.

The alpha diversity index did not show significant differences in richness—(i.e., the number of bacterial amplicon sequence variants [ASVs]) (Wilcoxon test; *P = *0.056) or evenness (inverse Simpson index) of the bacterial communities between branchiostegites from the black and brown color morphs (Wilcoxon test; *P = *0.127) (Fig. S5A). On the other hand, we found a slightly higher difference in richness (Wilcoxon test; *P = *0.010) between shrimp from the two vent fields, Kairei and Edmond, but there was no statistical significance in the evenness between the two (inverse Simpson index, Wilcoxon test; *P = *0.181) (Fig. S5B). Significant variations in the beta diversities of the bacterial communities on the branchiostegites of *R. kairei* were observed between the two vent fields (permutational analysis of variance [PERMANOVA]; *R*^2^ = 0.222, *P < *0.001) but not between the color morphs (*R*^2^ = 0.144, *P* = 0.0435). These results were further supported by hierarchal structuring and nonmetric multidimensional scaling (NMDS) ordination plots, which indicated some level of separation, albeit incomplete, between Kairei and Edmond samples but a lack of structuring between the color morphs (Fig. 4 and Fig. S6).

### Microbial taxonomic comparison.

**FIG 5:**
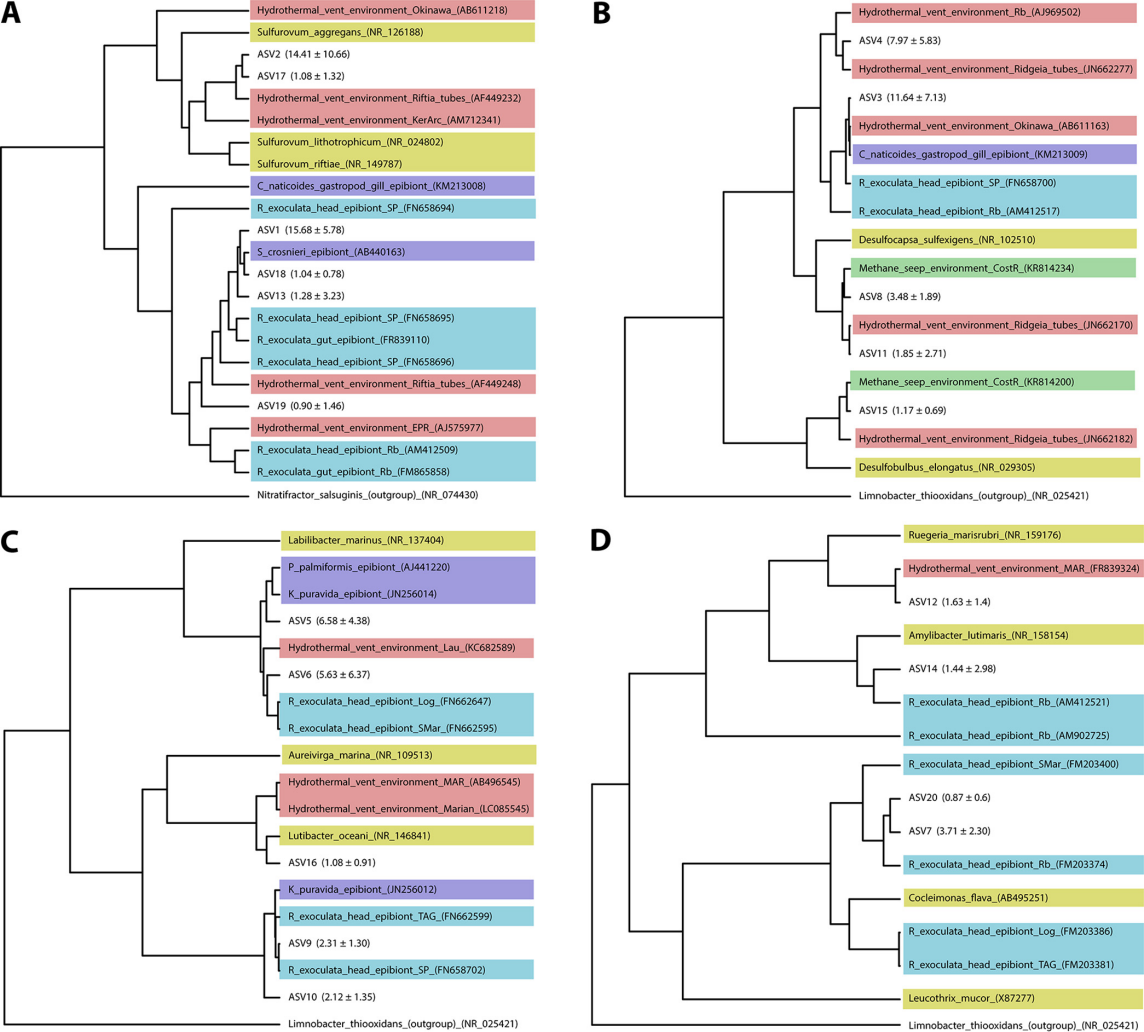
Phylogenetic analyses of the 20 dominant variants by Bayesian inference method, based on the general time-reversible (GTR) model. Colors are indicating the reference sequence origins. Numbers in brackets in front of ASVs indicate mean proportions ± standard deviation. Light blue, *Rimicaris exoculata* epibionts. Dark blue, vent fauna epibionts. Red, environmental bacteria from hydrothermal vents. Green, environmental bacteria from methane seeps. Yellow, cultivated bacterial strains. (A) Phylum *Campylobacterota*. (B) Phylum *Desulfobacterota*. (C) Phylum *Bacteroidota*. (D) Phylum *Proteobacteria*.

Metabarcoding of *R. kairei* branchiostegites from 13 individuals using the 16S partial rRNA (V4-V5 regions) revealed 129 unique ASVs affiliated with eight phyla based on the SILVA 138 database, with just four phyla accounting for over 99.8% of the sequences obtained. The most abundant group was Campylobacterota, representing 38.3% of the total data set in relative abundance, followed by Desulfobacterota (28.8%), Bacteroidota (23.4%), and Proteobacteria (9.3%) (Fig. 4). Overall, these bacterial communities were dominated by a limited number of ASVs, with the 20 most abundant ASVs representing 86% of the total data set in relative abundance. According to our phylogenetic reconstruction, many of these dominant variants were closely related to known symbionts of *R. exoculata* or R. chacei (Fig. 5). Nevertheless, others were more closely related to environmental strains from hydrothermal vents or methane seeps, in particular those from the *Desulfobacterota* phylum (Fig. 5).

Most ASV sequences were identified in both branchiostegite color morphs, with only 19 ASVs being specific to the black morphotype and three ASVs being specific to the brown morphotype (Fig. S7A). Similarly, a large proportion of the ASVs were common to both vent fields, with only 29 ASVs specific to Kairei and 10 ASVs specific to Edmond (Fig. S7B). A large part of this vent specificity resides at the variant level, as many vent-specific ASVs only differ by 1 to 3 nucleotides from an ASV specific to the other vent field or from an ASV shared by both (22 out of the 39 vent-specific ASVs). In addition, a few taxonomic groups were highlighted by our linear discriminant analysis (LDA) analysis as specific to one of the two vent fields (Fig. S8). Among them, variants affiliated with the phylum Verrumicrobiota, the class Desulfobacteria, and the two Gammaproteobacteria orders (Coxiellales and Granulosicoccales) were significantly more abundant in shrimp from Kairei. Variants affiliated with the Campylobacteraceae family, as well as several Bacteroidota genera, were also more represented at Kairei. In contrast, only bacteria affiliated with the genus Polaribacter were significantly more abundant in shrimps from Edmond.

### Fluorescent *in situ* hybridization.

**FIG 6:**
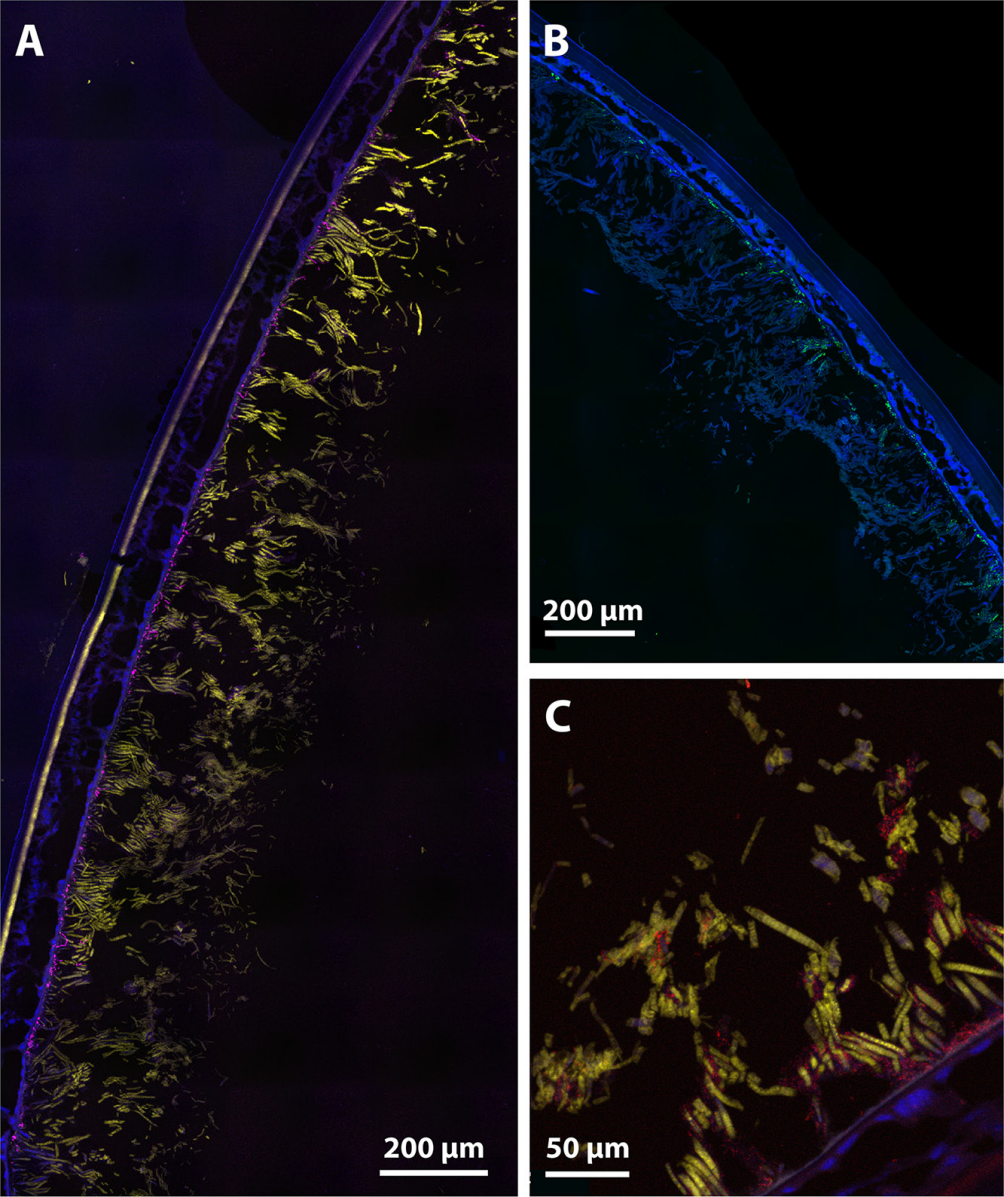
Fluorescent *in situ* hybridization (FISH) observations of *Rimicaris kairei* branchiostegites with specific probes performed on semi-thin sections (7 μm) stained with 4′,6-diamidino-2-phenylindole (DAPI) (blue). (A) Branchiostegite section of a black morph shrimp from Kairei hybridized with *Campylobacterota*-specific (yellow) and *Gammaproteobacteria*-specific (magenta) probes. (B) Branchiostegite section of a brown morph shrimp from Edmond hybridized with a *Desulfobacterota*-specific (green) probe. The upper right corner of this image was manually erased for illustrative purpose as it corresponds to the PAP pen circle; the raw image is available in Fig. S9 in the supplemental material. (C) Branchiostegite section of a black morph shrimp from Kairei hybridized with *Campylobacterota*-specific (yellow) and *Bacteroidota*-specific (red) probes.

Our FISH analyses showed that Campylobacterota taxa largely dominated the symbiont communities on the branchiostegites of *R. kairei* from both Kairei and Edmond fields and on both black or brown morphotypes (Fig. 6). These corresponded to a large portion of the thick and thin filamentous bacteria covering the inner side of the branchiostegites (Fig. 6A and C). A few other thin bacterial filaments hybridized with a *Gammaproteobacteria*-specific probe, although most of the positive signal observed with this probe corresponded to small cocci and rod-shaped bacteria on the basal part of the branchiostegites (Fig. 6A). Similarly, a positive signal was also obtained with Desulfobacterota-specific probes for small rod-shaped bacteria and cocci localized on the basal part of the branchiostegites (Fig. 6B). Finally, hybridizations with *Bacteroidota*-specific probes revealed that bacteria from this group were present and active within symbiotic communities of *R. kairei* as aggregations of several small cocci and rod-shaped bacteria attached to *Campylobacterota* filaments, on either the basal parts or the upper parts of the branchiostegites (Fig. 6C). No clear trends in terms of relative abundance could be established between the three other groups. Our observations did not reveal any variations in the spatial distribution of the dominant bacterial groups between the black and brown morphotypes.

## DISCUSSION

### Comparison with symbiotic communities from other *Rimicaris* species.

Our study confirmed the presence of dense bacterial communities on the inner face of *Rimicaris kairei* branchiostegites with a large morphological diversity, from rod-shaped to both thin and thick filamentous bacteria. This morphological diversity mirrors the observations from other Atlantic *Rimicaris* shrimps with well-characterized symbioses, including *R. exoculata* and *R. chacei* (18, 35), as well as Rimicaris hybisae (47), for which only SEM images have been published. Like *R. exoculata* and *R. chacei* (18, 35), the bacterial colonization on the branchiostegites of *R. kairei* was also limited to the anterior part, with the posterior part facing the gills being deprived of bacteria. The composition of mineral particles deposited on the branchiostegites of *R. kairei* was similar to that of the deposits observed in its sister species, *R. exoculata* (18, 48), with the presence in both morphotypes of iron oxyhydroxides in the form of ferrihydrite and of iron sulfide, identified as pyrrhotite in our study. We also note the presence of oxyhydroxides in the form of goethite, particularly in the brown morphotypes, and of methanethiol- or methionine-like organic matter in the black morphotypes.

The symbiont communities of *R. kairei* harbored a high phylogenetic diversity similar to that observed in its sister species *R. exoculata* (8, 14, 21–23, 27, 28). More specifically, we found a clear dominance of *Campylobacterota* symbionts (14, 21, 23, 27, 28), which constituted most of the thin and thick filamentous bacteria on the surface of *R. kairei* branchiostegites. We also observed the presence of *Gammaproteobacteria*, although to a lower extent than that in *R. exoculata*, mostly in the form of rod-shaped bacteria and with fewer thin filaments than observed in *R. exoculata* (14, 21, 23, 27). *Desulfobacterota* (formerly known as Deltaproteobacteria [49]) taxa were also abundant, mostly distributed on the basal part of branchiostegites, close to the surface. Additionally, we confirmed the presence of *Bacteroidota* as active bacteria constituting an important component of the *R. kairei* symbiotic community. These were found to be aggregations of small cocci or rod-shaped bacteria on and around the *Campylobacterota* filaments. This location supports the hypothesis of a syntrophic relationship between *Bacteroidota* and *Campylobacterota* symbionts on *Rimicaris* branchiostegites, similar to such relationships shown in biofilm communities from the Loki’s Castle vent field (50).

According to our phylogenetic analyses, most of the dominant variants from these four bacterial phyla on the *R. kairei* branchiostegites were closely affiliated with *R. exoculata* epibionts (Fig. 5), suggesting a similar core symbiont composition between the two sister species across Indian and Atlantic Oceans. Still, *Desulfobacterota* symbionts of *R. kairei* were more diverse than previously reported for *R. exoculata*, with variants more closely related to environmental lineages reported from both hydrothermal vents (51) and methane seeps (52). Although our 16S metabarcoding analyses appeared to indicate higher relative abundances of *Desulfobacterota* and *Bacteroidota* over *Gammaproteobacteria* taxa, these trends could not be clearly confirmed with our FISH observations. Such differences could have arisen from methodological issues, including amplification bias or mismatches impairing probe hybridization on the 16S rRNA for some lineages. We also cannot exclude the possibility that these discrepancies between our FISH and metabarcoding results could be related to the presence of dormant or inactive lineages in these communities at the time our samples were processed and fixed for FISH observation.

Overall, these results support a trophic role of *R. kairei* cephalothoracic symbionts, already suggested by stable isotope ratios (39) and the modified anatomy of these shrimp, i.e., enlarged cephalothorax and hypertrophied mouthparts (53). Therefore, a direct nutritional transfer from symbionts to their host through the cephalothorax integument in a similar fashion to that in *R. exoculata* (4) can be assumed for *R. kairei*, given the proximity of the two shrimp both anatomically and phylogenetically.

### Symbiont community composition is independent of color or mineralization.

We found that elemental state and mineralogical composition within the *R. kairei* cephalothoracic cavity (i.e., the inner surface of the carapace) differed considerably between black and brown morphs. Whereas the mineral deposit in the brown morph was mostly composed of goethite, with a lower proportion of ferrihydrite, in the black morph, ferrihydrite was the most abundant mineral, followed by pyrrhotite and goethite in lower proportions (Fig. 4). In addition to those mineral compositions, the reduced state of sulfur is contained in the organic matter in the black morph. This variation in mineral composition and elemental state likely reflects the precipitation of reduced compounds from vent fluids with different compositions and/or their transformation by symbiotic partners with different metabolic pathways. Surprisingly, our results revealed that the symbiotic community composition on the branchiostegites of *R. kairei* did not differ according to the color morph of their cephalothorax (Fig. 3). Although limited, a greater difference was observed between shrimp from the two hydrothermal fields, regardless of the color of their cephalothorax. These results contrast with previous work on *R. exoculata*, where black shrimp were found to host distinct symbiont communities compared to those of the red morphotype (14). Part of the answer may lie in the high metabolic flexibility of *R. kairei* symbionts. Although prediction of functional profiles from 16S partial rRNA data is often misleading, the metabolic capacities of *R. kairei* symbionts can be predicted with reasonable accuracy based on those observed in *R. exoculata* symbionts, owing to their close phylogenetical and ecological proximity.

Several metagenomic studies have pointed out the potential of *R. exoculata*, *Campylobacterota*, and *Desulfobacterota* symbionts to use either hydrogen or diverse reduced sulfur compounds as an energy source through oxidation or fermentation reactions (8, 27, 28). It has been shown that hydrogen is a particularly favorable electron donor whose energetic yield is far greater than that of other reduced compounds, providing up to 18 times more energy per kilogram of vent fluid than H_2_S in other chemosynthetic symbioses (5, 54). Therefore, under H_2_-rich environmental conditions like those of the Kairei vent field (43, 44), *Campylobacterota* and *Desulfobacterota* symbionts probably preferentially use H_2_ over H_2_S as their main energy source (8, 27, 28). The H_2_S concentrations in the vent fluids of Kairei and Edmond have been measured previously, showing that they are in the same order of magnitude (43, 44). In this context, a higher proportion of the H_2_S pool is not used by the symbiont metabolisms and could react with the iron from the fluid, increasing the formation of pyrrhotite characteristic of the black morph shrimp. This would explain the dominance of the black morph phenotype at Kairei, the fluids of which are among the richest in H_2_ among all known vents (43, 44). On the other hand, under hydrogen-poor conditions like at Edmond, the *Campylobacterota* symbionts would switch their metabolism to sulfur oxidation using the H_2_S available pool from the fluid, leading to the formation of sulfur compounds with various degree of oxidation from SO_4_^2−^ to elemental sulfur (27). This phenomenon has been shown experimentally for the Alviniconcha marisindica snail symbiosis with *Campylobacterota* endosymbionts in the Kairei and Edmond fields (55), and the same likely occurs in *R. kairei*. In addition, *Desulfobacterota* symbionts can also reconstitute part of this H_2_S pool through sulfur disproportionation by using some of the oxidized sulfur compounds produced by *Campylobacterota* metabolism as both electron donors and acceptors in an overall syntrophic sulfur cycle (28). Whether pyrrhotite develops or not would result in this case from a subtle equilibrium between these different sulfur compound ratios that could be available under certain conditions to react with the iron pool, leading to the presence of both brown and black morphotypes at this vent field. The same scenario could also be taking place in *R. exoculata* at the Trans-Atlantic Geotraverse (TAG) vent field, which harbors similar iron concentrations compared to those at Edmond (43, 56) and where both black and red/brown morphotypes of *R. exoculata* have been observed (20, 41, 42). In these cases, changes in the dominant metabolic pathways do not reflect changes of partner composition within the symbiotic system, and in such cases the same microbial community may produce different mineralogy under different environmental factors.

In *R. exoculata* shrimps from the Logatchev and Rainbow vent fields on the MAR, variations in vent fluid composition (56) have been shown to influence the composition of *R. exoculata* symbiotic communities (14). Unlike the Indian Ocean fields, both Logatchev and Rainbow exhibit high concentrations of methane (CH_4_) in addition to high concentrations of H_2_ (43, 44, 56) but differ in their iron concentration, which is extremely high at Rainbow and low at Logatchev (56). Both methane and iron oxidations exceed the metabolic capacities of *Campylobacterota* and *Desulfobacterota* symbionts and are carried out by methanotrophic *Gammaproteobacteria* and iron-oxidizing *Zetaproteobacteria*, respectively, in the *R. exoculata* symbiosis (8, 27). Therefore, a switch of microbial metabolisms corresponds here to a change in the abundance of the different bacterial partners (14).

Although an absence of methanotrophic symbionts could be anticipated in our populations of *R. kairei* given the low CH_4_ concentrations at Kairei and Edmond (43, 44), the absence of iron-oxidizing *Zetaproteobacteria* was unexpected. Rather than the vent fluid chemistry itself, this absence of *Zetaproteobacteria* in the *R. kairei* symbiotic communities could be related to other unsuitable environmental conditions in the Indian Ocean vents preventing the association between the two—or to biogeographical barriers for the specific *Zetaproteobacteria* lineages associated with *Rimicaris exoculata* on the MAR. Like other cephalothoracic epibionts, *Zetaproteobacteria* in *Rimicaris* shrimps are supposedly acquired horizontally, and their presence in these symbiotic communities thus requires the presence of a local environmental pool (14, 57). Microbial mats communities with abundant *Zetaproteobacteria* have been reported in several Atlantic vents (58), the Lau Basin, and the Tonga Arc (59, 60), as well as in the Mariana Trough, Mariana Arc (60–62), and at the Loihi seamount near Hawaii (63). To our knowledge, *Zetaproteobacteria* taxa have not yet been reported from either the Kairei or Edmond fields on the Central Indian Ridge (CIR), although they have been reported from a vent on the Southwest Indian Ridge (64). Nevertheless, the presence of the specific *Zetaproteobacteria* lineages associated with *R. exoculata* remain undetected on the CIR.

Our observations of ferrihydrite deposits in abundant quantities on the branchiostegites of *R. kairei* remains surprising, as they were interpreted as a sign of biogenic iron-oxidizing activity for *R. exoculata* (40, 41). The presence of such ferric oxides and sheaths has also been observed associated with *Zetaproteobacteria* on the surface of *R. exoculata* egg broods, where this lineage was particularly abundant (57). Similarly, iron oxyhydroxide in the form of goethite has also sometimes been attributed directly to microbial activities or to the ageing of microbe-produced ferrihydrite to more crystalline forms (65), although it often results from abiotic iron oxidative processes as well. Even if such iron deposits could arise from abiotic processes of iron oxidation in *R. kairei* in Indian Ocean vents, unlike in *R. exoculata* in Atlantic vents, we also cannot exclude the possibility that iron oxidation is being performed by non-*Zetaproteobacteria* lineages in *R. kairei*. Biogenic iron oxidation in marine bacteria has also been reported in some *Gammaproteobacteria* and *Alphaproteobacteria* taxa (59, 66). Among the most abundant ASVs found on *R. kairei* branchiostegites, one of the two ASVs affiliated with *Alphaproteobacteria* was distinctive and not closely related to any known *R. exoculata* symbiont (Fig. 5). Further investigations comparing the mineralogical compositions and the metabolic capacities of *R. kairei* with *R. exoculata* cephalothoracic symbiont communities through metagenomic analyses, as well as *in situ* measurements of environmental conditions in the shrimp colonies, will help disentangle the respective influences of local environmental conditions and biogeographic barriers in these vent shrimp symbiotic communities.

## MATERIALS AND METHODS

### Sample collection.

*Rimicaris kairei* shrimps were collected on the Central Indian Ridge (CIR) at two vent fields, Kairei (25°19.2253′S, 70°2.4217′E, depth 2,415 m) and Edmond (23°52.6823′S, 69°35.8013′E, depth 3,290 m) (Fig. 1A), during the YK16-E02 expedition (4 February to 2 March 2016) onboard R/V *Yokosuka*. Specimens were collected from large swarms inhabiting actively venting black-smoker chimneys (Fig. 1B and C), using a suction sampler on the Human-Operated Vehicle (HOV) *Shinkai 6500*. Upon recovery on the ship, shrimp were frozen at −80°C or preserved by 99.5% ethanol for further analysis. The shrimps were identified as black morph (Fig. 1B) and brown morph (Fig. 1C) according to the coloration of their branchiostegites (Br) in the laboratory. Overall, the black morph was dominant at Kairei, whereas both black and brown morphs were found at Edmond (Fig. 1).

### Scanning electron microscopy.

Four brown morph *R. kairei* from Edmond and three black morph shrimps from Kairei preserved in ethanol were used for SEM observations. Shrimp were dissected and branchiostegites were separated for further analysis. After brief observations using a dissecting microscope (SZX7; Olympus), the dissected parts were put into *t*-butyl alcohol and frozen. The frozen samples were critical point dried under vacuum conditions. The dried samples were then coated by gold using ion sputter JFC-1200 (JEOL) and observed using SEM (JSM-6100LV; JEOL) and field emission (FE)-SEM (JSM-7100F; JEOL).

### Mineralogical analyses.

To investigate the distribution pattern and relationships of microbes and minerals in the gill chamber of *Rimicaris*, thin sections were made using ethanol-preserved *R. kairei* specimens. The shrimp were placed into Spurr resin. The shrimp stayed under vacuum condition at room temperature for half a day for permeation of the resin. The process of Spurr resin permeation was repeated four times for complete permeation. Then, the resin with shrimp was heated at 60°C under vacuum conditions for 48 h for thermal polymerization of the resin. Hardened resin with shrimp was cut into chips using a diamond saw. The chips were put onto slide glasses and polished using a graded series of silicon carbide powders and 1-μm-size diamond powders. The thin sections were observed under a polarizing optical microscope (Eclipse LV100POL; Nikon) and a field emission scanning microscope (JSM-7100F; JEOL). Elemental analysis was performed using the same FE scanning electron microscope equipped with E-MAXn/WAVE (Oxford Instruments). For SEM and elemental analysis, thin sections were coated by carbon using a quick carbon coater (SC-701CT; Sanyu Electron). In total, seven shrimp specimens, four brown from the Edmond site and three black from the Kairei site, were used for SEM observations and EDX analyses. Two individuals, one for each color morph, were used for further micro-X-ray analysis.

Elemental redox states were determined by X-ray absorption near edge structure (XANES) analysis using a micro-X-ray beam at a synchrotron facility at the Photon Factory of the High Energy Accelerator Research Organization (KEK-IMSS-PF) in Tsukuba, Ibaraki Prefecture, Japan. Iron and sulfur μ-XANES spectra were recorded at the BL-4A and BL-15A beamlines, respectively, both equipped with an Si(111) double crystal monochromator. At the BL-4A beamline, the thin sections were set on a sample holder oriented at 45° to the beam. The beam was focused by a Kirkpatrick-Baez (K-B) mirror, and the beam size was 5 μm by 5 μm on the sample. The Fe μ-XANES spectra of reference materials and the *R. kairei* sample were measured in transmission and fluorescence modes, respectively. Fluorescence X-rays (XRF) were detected by a single-element silicon drift detector (SDD). At the BL-15A1 beamline, the thin sections were set on a sample holder oriented at 45° to the beam. The beam size was 20 μm by 20 μm. The S μ-XANES spectra of reference materials and the *R. kairei* sample were both measured in fluorescence mode. Fluorescence X-rays (XRF) were detected by a single-element SDD. The energy calibrations for Fe and S were performed using reference material for each. Analytical points for XANES spectra for both Fe and S were determined by the μ-XRF maps of the shrimp samples using 12-keV and 4.2-keV incident X-ray, respectively. The μ-XANES spectra of Fe and S were processed using data analysis software package REX2000 (Rigaku).

### DNA extraction and sequencing.

Seven frozen (−80°C) *R. kairei* shrimp from Edmond (three black and four brown) and six *R. kairei* shrimp from Kairei (five black and one brown) were used for DNA extraction. A PowerSoil DNA isolation kit was used to extract DNA from branchiostegites of *R. kairei* shrimp following the manufacturer’s instructions (MO Bio Laboratories, Inc., Carlsbad, CA). Concentrations of extracted DNA varied from 0.85 to 62.1 ng/mL. Samples with DNA concentrations higher than 20 ng/mL were diluted to 10 ng/mL. The V4-V5 variable region of 16S rRNA genes were amplified with LA *Taq* polymerase (TaKaRa Bio, Kusatsu, Japan) in 35 cycles for samples with DNA concentration lower than 10 ng/mL or in 30 cycles for those diluted to 10 ng/mL, using the universal primer set of U530F and U907R (67) under the following PCR conditions: 20 s at 96°C, 45 s at 52°C, and 1 min at 72°C. The PCR products were then purified using Exo-SAP-it (Affymetrix) according to the manufacturer’s instruction. The amplicons were mixed with PhiX control libraries and sequenced on an MiSeq platform (Illumina, San Diego, CA) at the Japan Agency for Marine-Earth Science and Technology (JAMSTEC), Yokosuka, Japan. Due to the challenges of sample collection in hydrothermal vents, we did not include a control sample from the vents.

### Sequencing processing.

A total of 1,303,090 raw reads across 13 samples, averaging 100,238 reads per sample, were analyzed using the DADA2 pipeline (68). Sequences were truncated to 260 bp for forward reads and to 210 bp for reverse reads based on the average quality scores. Additionally, reads displaying “N”, a quality score below 2, and/or more than 2 expected errors were discarded. The error model was trained using 1,000,000 sequences before denoising, and chimeric sequences were removed based on a consensus approach before the paired ends were assembled.

The final data set contained 1,055,624 reads, with an average of 81,202 sequences per sample after quality filtering. Representative sequences were classified into taxonomic groups using the SILVA 138 database (69). Additional filtering on abundance was conducted at a threshold of 0.005% (70) to remove sequences containing non-biologically-relevant amplicon sequence variants (ASVs; singletons and doubletons mostly resulting from sequencing errors and are not part of the real communities). Those ASVs affiliated with mitochondria sequences of the shrimp host were also manually removed from the data set.

### Statistical analyses.

Alpha diversity within the 13 samples was estimated with ASV number and inverse Simpson index (71). Difference in the alpha diversity indexes among color and vent field categories were tested using Mann-Whitney tests; a *P *value of <0.05 was considered the threshold of statistical significance for a difference between samples.

For further statistical analysis, the sequence data set was normalized by cumulative sum scaling to minimize the effect of different sequence numbers obtained with each sample (72). Beta diversity was analyzed by nonmetric multidimensional scaling (NMDS) ordination and by hierarchical clustering using the complete method based on Bray-Curtis dissimilarity matrices. The homogeneity between categories was tested with the “betadisper” function of the *vegan* R package, and significant differences between categories were tested for by permutational analysis of variance (PERMANOVA; 9,999 permutations) with the “adonis” function of the same package (73). Taxonomic composition and diversity results were visualized using the R package *Phyloseq* v. 1.14.0 (74).

The linear discriminant analysis (LDA) effect size (LefSE) method (75) was used to characterize and highlight microorganisms specific to each of the different conditions. Results were shown with a cladogram, where each concentric circle represents a taxonomic level, the innermost being the phylum and the outermost being the genus.

### Fluorescent *in situ* hybridization.

Two frozen shrimp individuals—one for each color morph, a black one from Kairei and a brown from Edmond—were directly placed into 4% paraformaldehyde (PFA) and fixed for 3 h, then stored in 50% phosphate-buffered saline (PBS)-ethanol solution in the laboratory. Branchiostegites were dissected and rehydrated in a PBS-ethanol series from 50/50 to 1× PBS with a 25% increase for each bath of 15 min each. Branchiostegites pieces were then progressively transferred to an optimum temperature compound (OCT) (Tissue-Tek; Sakura Finetek, Osaka, Japan) with a first bath in 15% sucrose-PBS, followed by a bath in 30% sucrose-PBS and a final bath in 30% sucrose-PBS-OCT, for 30 min each, before embedding in OCT in a plastic holder at 4°C.

The OCT-embedded branchiostegites pieces were cross-sectioned at 7-μm thickness using a cryostat (CM1520; Leica Biosystems) at a chamber temperature of −20°C. Individual sections were thaw-mounted onto adhesive glass slides (76 mm by 26 mm; Matsunami, Osaka, Japan). Prior to *in situ* hybridization, sections were washed two times in 1× PBS buffer for 5 min, postfixed in 1× PBS-10% formalin for 10 min, and washed again three times in 1× PBS for 5 min.

Sections were hybridized in a reaction mixture containing 0.5 mM each probe in a 35% formamide hybridization buffer (0.9 M NaCl, 0.02 M Tris-HCl, 0.01% sodium dodecyl sulfate [SDS], and 35% deionized formamide) for 3 h at 46°C. Hybridization temperature was chosen according to a published protocol (14). Sections were washed at 48°C for 30 min in a washing buffer (0.102 M NaCl, 0.02 M Tris-HCl, 0.005 M EDTA, and 0.01% SDS) and rinsed briefly with water, air dried, and covered with SlowFade Gold antifade reagent mounting medium containing 4′,6-diamidino-2-phenylindole (DAPI; Invitrogen) and a cover slip. The probes used in this study (Fasmac) were Epsy549, targeting *Campylobacterota* (76); Dsb706, targeting *Desulfobacterota* (77); Gam42a, targeting *Gammaproteobacteria* (78), and Cfb563, targeting *Bacteroidetes* (79), labeled with cyanine 3 or cyanine 5 (see Table S1 in the supplemental material). Observations were made using an A1RMP confocal scanning system (Nikon Instech, Tokyo, Japan) and processed using the NIS-Elements software (Nikon Instech).

### Data availability.

The 16S rRNA data are available in the NCBI SRA repository (submission identifier SUB11019173 and BioProject identifier PRJNA802196).
