# Supplementary material for: Symbiont Community Composition in Rimicaris kairei Shrimps from Indian Ocean Vents with Notes on Mineralogy
Source: Appl Environ Microbiol. 2022 Apr 11;88(8):e00185-22. doi: 10.1128/aem.00185-22 (PMC9040608; doi:10.1128/aem.00185-22)
Supplement: Supplemental file 1 — Fig. S1 to S9. Download aem.00185-22-s0001.pdf, PDF file, 1.3 MB [file aem.00185-22-s0001.pdf]

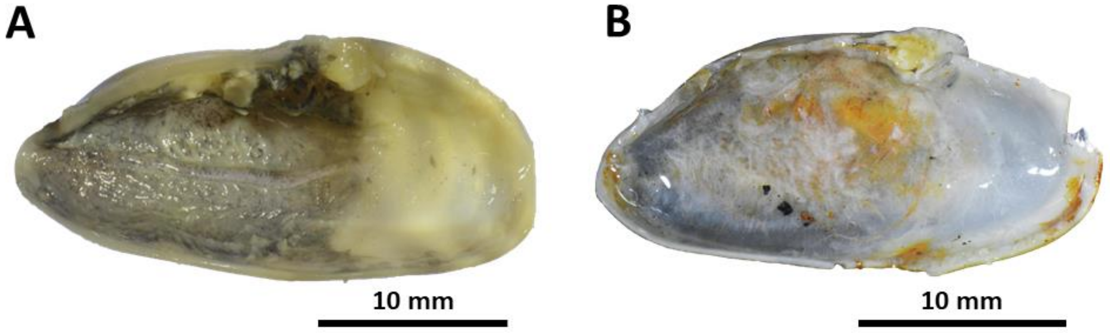

Figure S1. Dissected branchiostegites of *Rimicaris kairei* shrimps. **A.** from a black morph shrimp. **B.** from a brown morph shrimp.

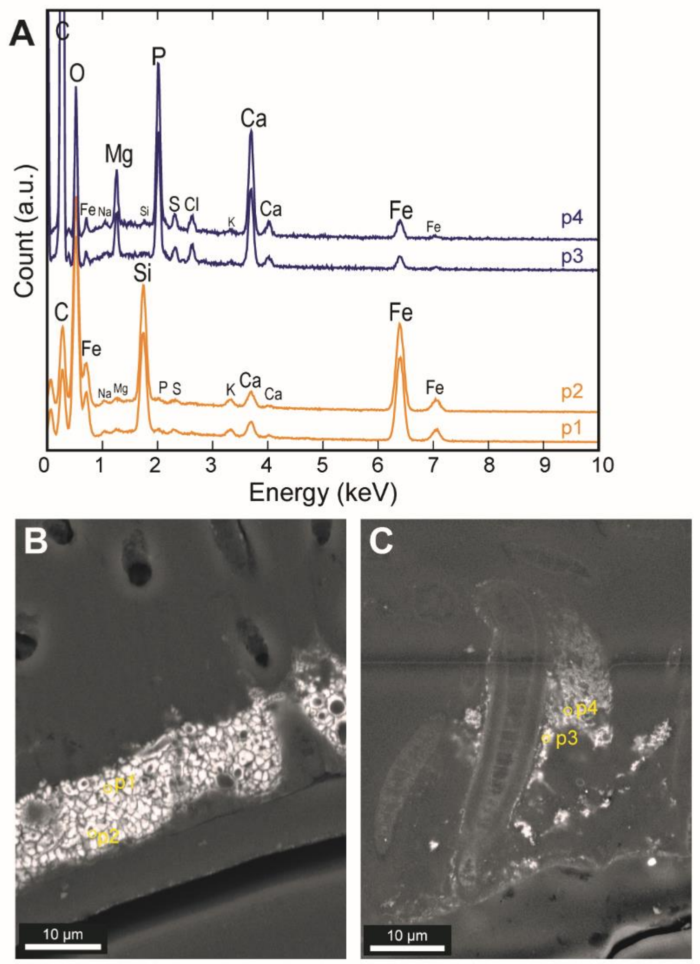

Figure S2. A. EDS spectrum of crusts on the internal surface of branchiostegites of brown *Rimicaris kairei* from Edmond Field (orange) and sheath of filamentous bacteria on branchiostegites of black *R. kairei* from Kairei Field (purple). **B.** Back scattered image showing crusts on branchiostegite of a brown shrimp. Analytical points were p1 and p2. **C.** Filamentous bacteria with sheath on branchiostegites of a black shrimp. Analytical points were p3 and p4 on the sheath.

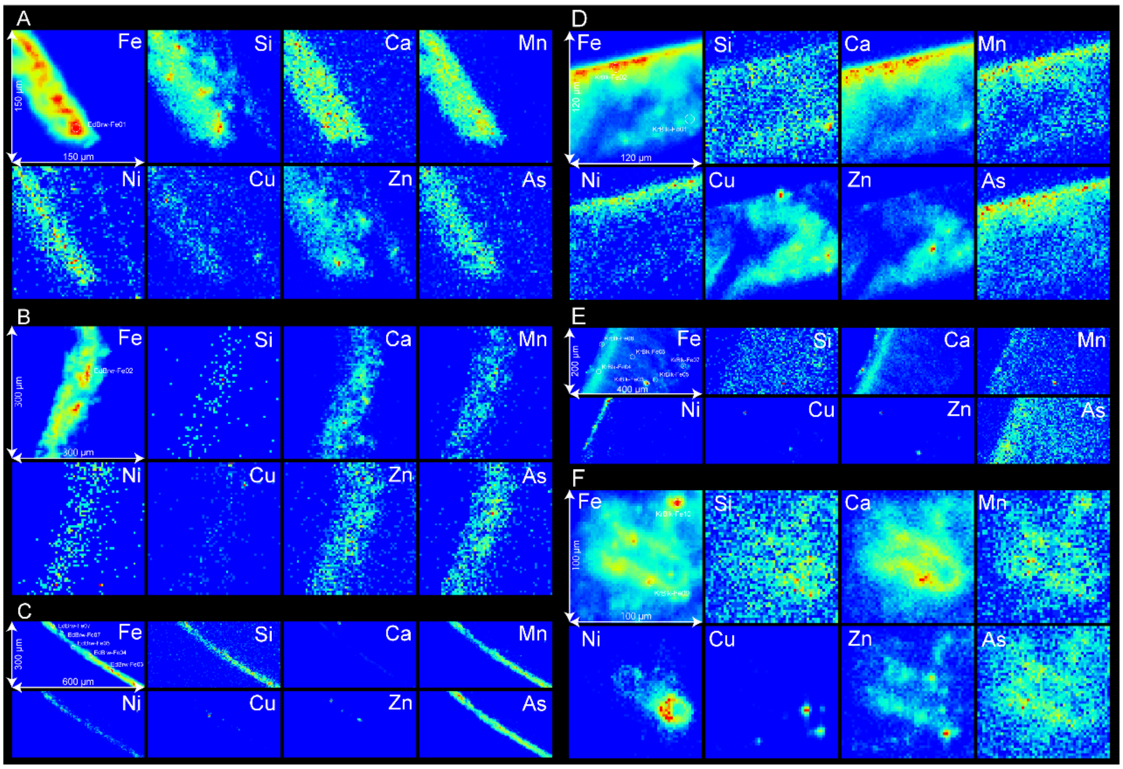

Figure S3. Analytical points (white open circles) of Fe K-edge XANES analyses for iron showing on XRF map images. Brown particles on branchiostegites of the brown morph from Edmond field (A-C). Black particles on the branchiostegites (D, E) and the maxilliped setae (F) of the black morph from Kairei field.

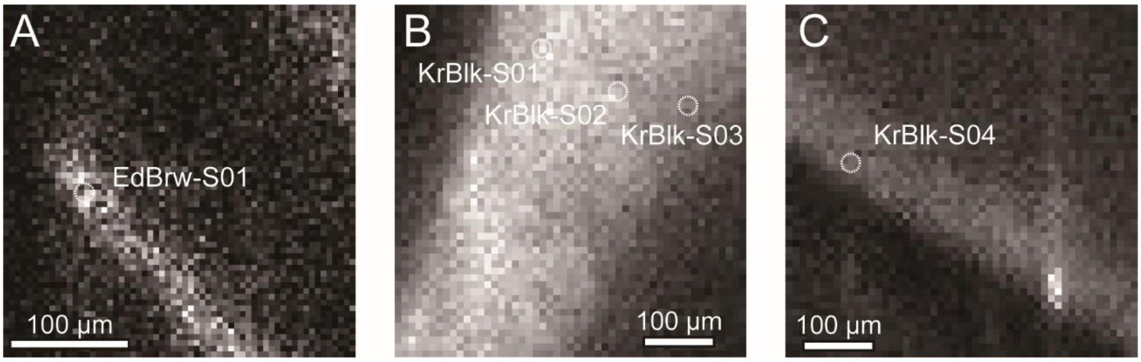

Figure S4. Analytical points (white open circles) of S K-edge XANES analyses for sulfur showing on XRF map images of iron. Brown particles on branchiostegites of the brown morph from Edmond field (A). Black particles on branchiostegites of the black morph from Kairei field (B, C).

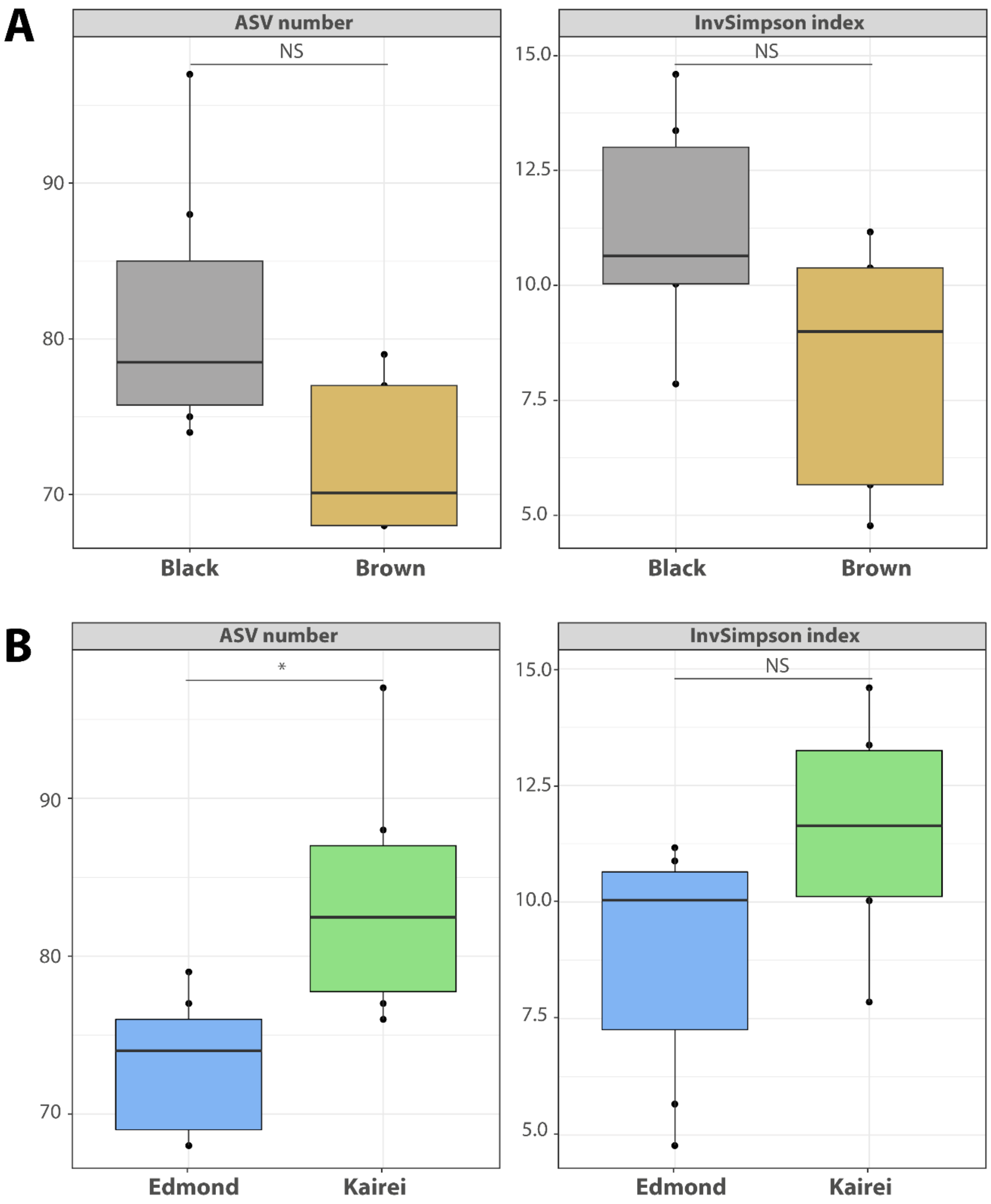

Figure S5. Alpha Diversity analysis. **A.** ASV number and Inverse Simpson index compared between brown and black shrimps. **B.** ASV number and Inverse Simpson index compared between shrimps from Edmond and Kairei

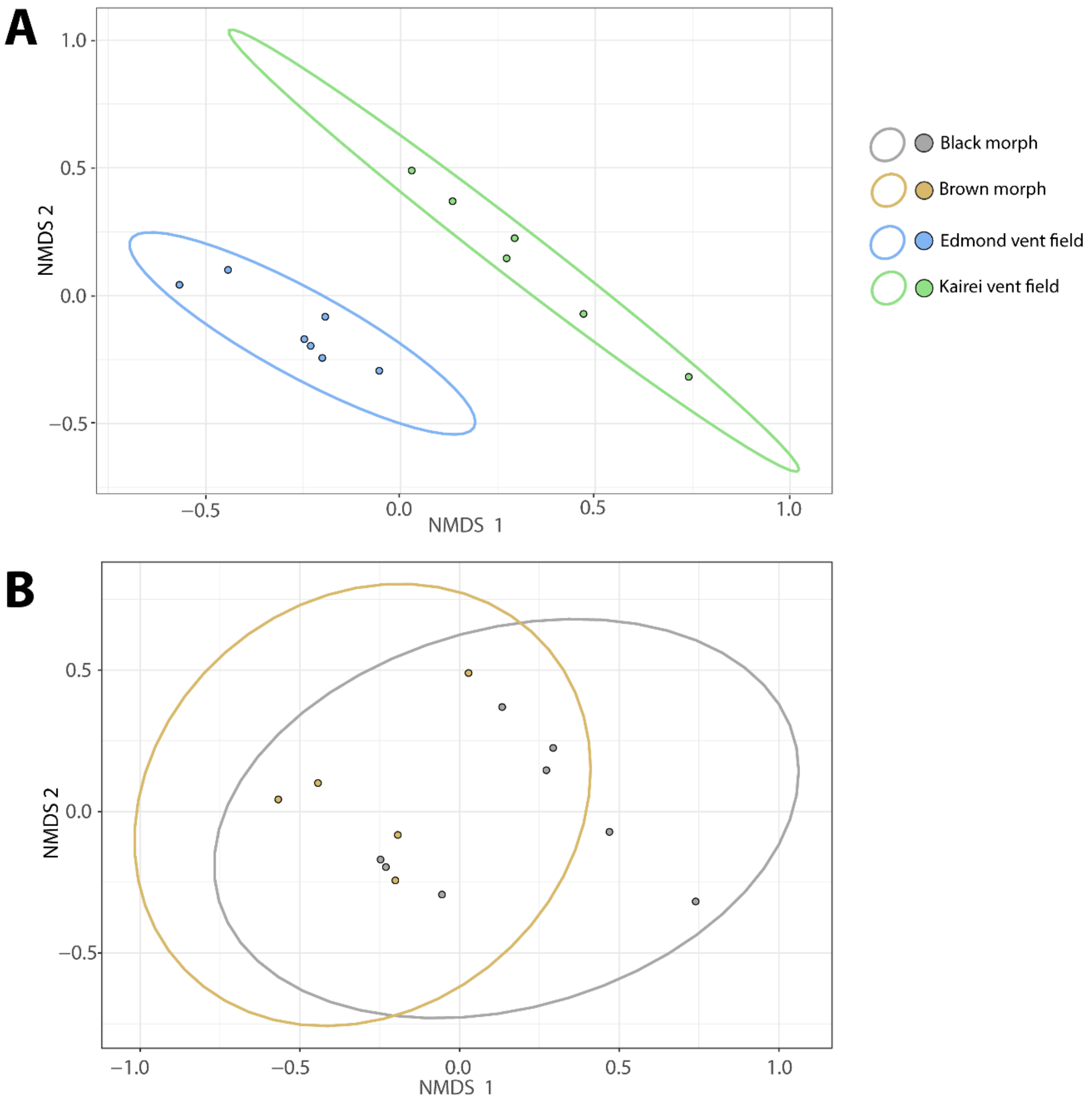

Figure S6. NMDS biplot of a Bray–Curtis dissimilarity matrix of the ASV relative abundance data

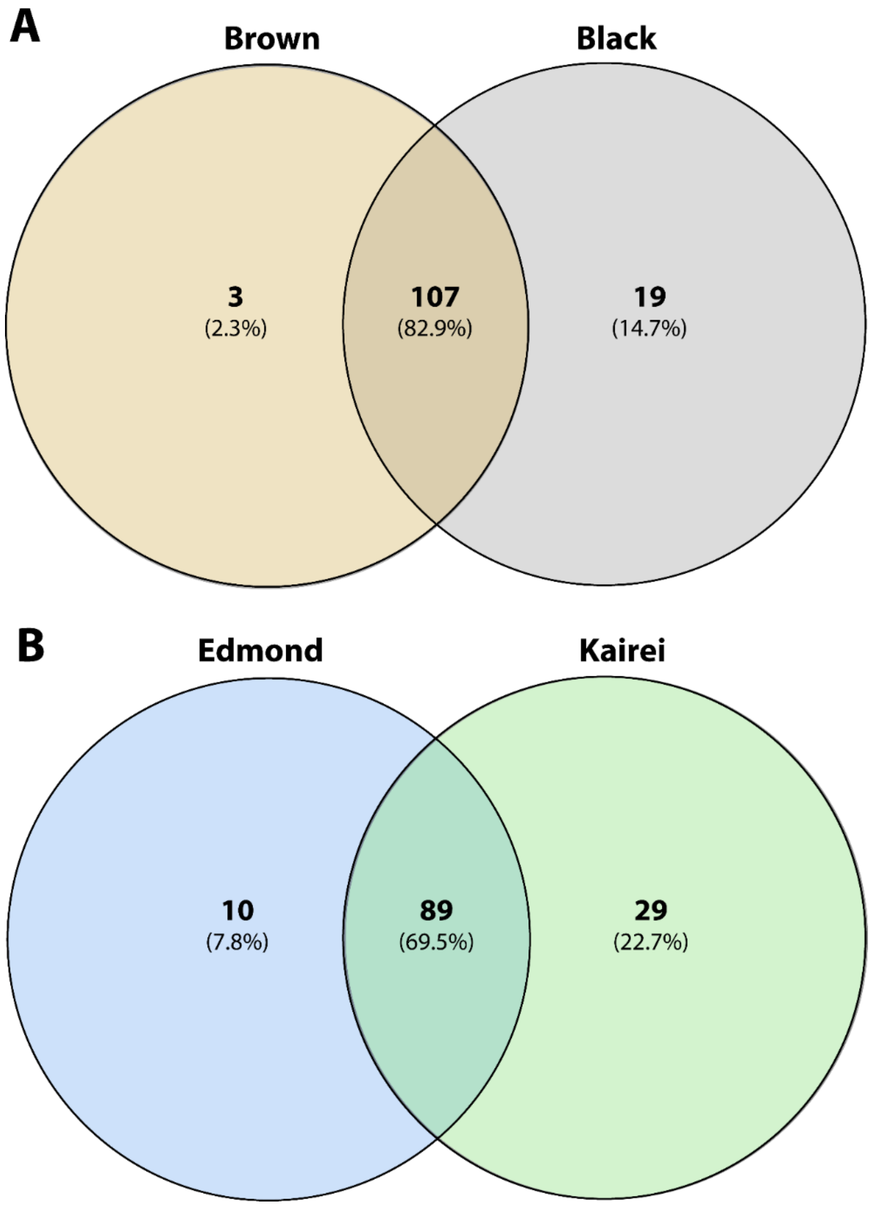

Figure S7. Venn diagrams. **A.** depicting ASVs shared between branchiostegite colors and ASVs specific to one color. **B.** depicting ASVs shared between vent fields and ASVs specific to one vent field.

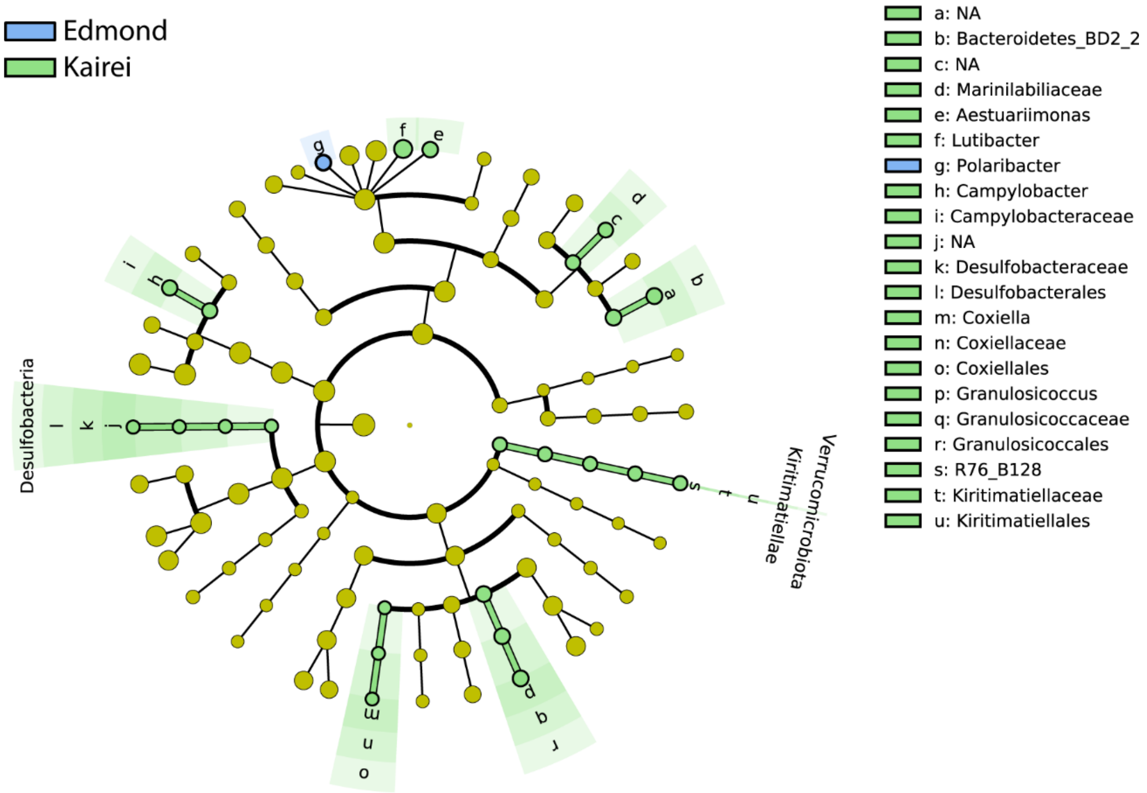

Figure S8. Cladogram representing taxonomic distribution of the differentially abundant bacterial taxa. The dots represent the specific bacterial taxa, with colors corresponding to the vent fields (Kairei or Edmond) where these are relatively more abundant. Yellow-greenish color denoting taxa with no significant difference in relative abundance between Kairei and Edmond fields. The size of dots is proportional to the relative abundance.

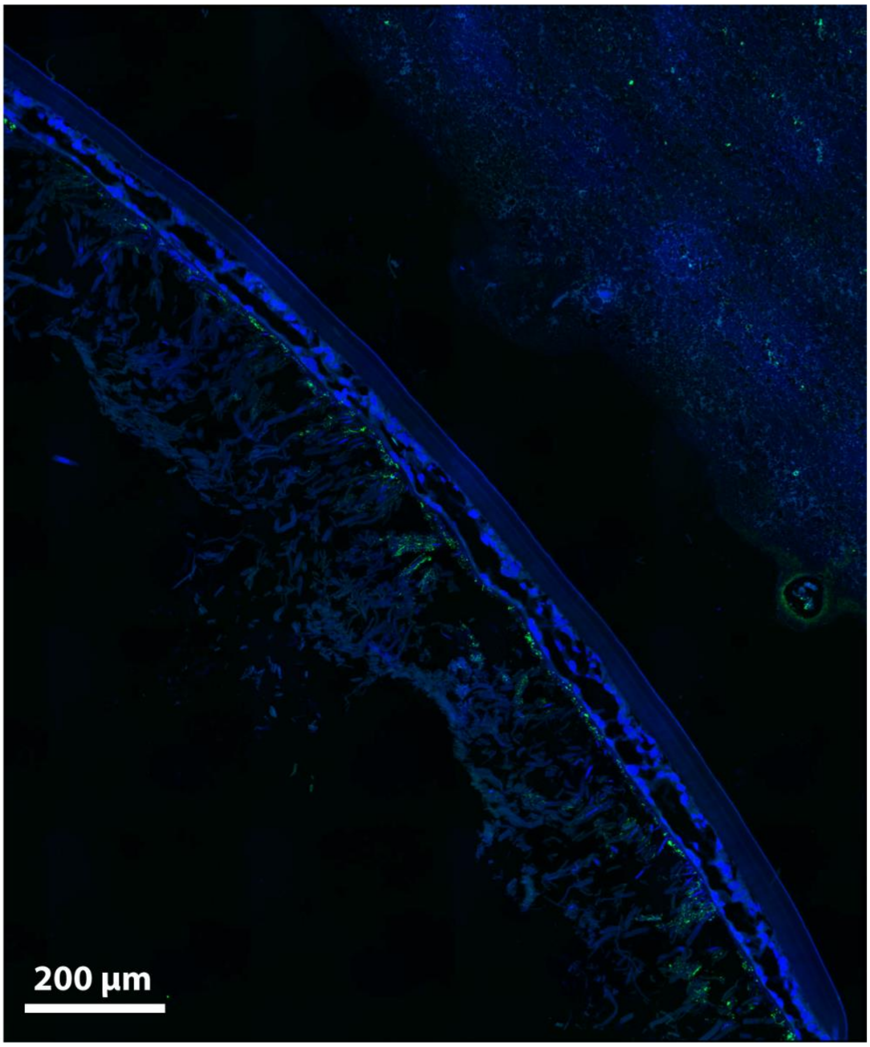

Figure S9. Raw image of FISH observation made on a branchiostegite section of a brown morph shrimp from Edmond hybridized with *Desulfobacterota* (green) specific probe.
